# Improving Hip Fracture Care in Ireland: A Preliminary Report of the Irish Hip Fracture Database

**DOI:** 10.1155/2014/656357

**Published:** 2014-12-08

**Authors:** Prasad Ellanti, Breda Cushen, Adam Galbraith, Louise Brent, Conor Hurson, Emer Ahern

**Affiliations:** ^1^Department of Trauma and Orthopaedics, St. Vincent's University Hospital, Elm Park, Dublin 4, Ireland; ^2^Department of Geriatric Medicine, St. Luke's General Hospital, Kilkenny, Ireland; ^3^Department of Nursing, Waterford University Hospital, Waterford, Ireland

## Abstract

*Introduction*. Hip fractures are common injuries in the older persons, with significant associated morbidity and mortality. The Irish Hip Fracture Database (IHFD) was implemented to monitor standards of care against international standards. *Methods*. The IHFD is a clinically led web-based audit. We summarize the data collected on hip fractures from April 2012 to March 2013 from 8 centres. *Results*. There were 843 patients with the majority being (70%) female. The 80–89-year age group accounted for the majority of fractures (44%). Most (71%) sustained a fall at home. Intertrochanteric fractures (40%) were most common. Only 28% were admitted to an orthopaedic ward within 4 hours. The majority (97%) underwent surgery with 44% having surgery within 36 hours. Medical optimization (35%) and lack of theatre space (26%) accounted for most of the surgical delay. While 29% were discharged home, 33% were discharged to a nursing home or other long-stay facilities. There was a 4% in-hospital mortality rate. *Conclusions*. Several key areas in both the database and aspects of patient care needing improvement have been highlighted. The implementation of similar databases has led to improved hip fracture care in other countries and we believe this can be replicated in Ireland.

## 1. Introduction

The Irish Hip Fracture Database (IHFD), the first of its kind in Ireland, is a national clinical audit developed to improve hip fracture care and outcomes in Ireland. Through the synergy of audit, clinical standards, and feedback it aims to provide a nationwide platform upon which each individual service can effectively measure, compare, and ultimately improve its service provision.

In 2008, a national report on falls and fracture in Ireland's ageing population identified hip fractures as one of the most serious injuries due to a fall, resulting in lengthy hospital admissions, ongoing care in step-down facilities, and ultimately a high cost to the health service [1]. The rate of hip fracture in the total population aged 50 and over was 407 for females and 140 for males per 100,000 [2]. Furthermore, hip fractures account for half of all fractures in patients over the age of 65 [3]. With approximately 3,000 people sustaining hip fractures on an annual basis in Ireland, a figure which will inevitably increase given our ageing population, hip fractures, and the management thereof presents a significant challenge for both geriatricians and orthopaedic surgeons alike.

The implementation of a hip fracture database has been shown to improve the quality of care of hip fracture patients in other countries [4]. The Swedish National Hip fracture Registry, Rikshöft, was established in 1988 to capture hip fracture related data [5]; this was soon followed by the Scottish Hip Fracture Audit (1993–2008). In 2007, the British Orthopaedic Association (BOA) and British Geriatrics Society (BGS) published the Blue Book,* The Care of Patients with Fragility Fractures* [6], which describes six quality care standards derived from evidence-based clinical practice. Using these care standards, The National Hip Fracture Database (UK NHFD) in the United Kingdom (excluding Scotland) has done much to improve care in the area since its establishment in 2007 [7] and through repeated audits has driven annual improvement in hip fracture care. Other established databases are NOREPOS Hip fracture Database in Norway [8] and NORM hip fracture registry in Malaysia [9]. Similar databases are in various stages of development in Australia, New Zealand, and Canada.

Using the key quality indicators set out in the Blue Book, the goal of the IHFD is to ensure that all patients admitted to hospital with a hip fracture receive high quality surgical management of the hip fracture, high quality acute medical management including effective secondary falls and fracture prevention, and high quality rehabilitation after fracture. Through the IHFD we hope to measure our care against these international standards and determine areas for improvement on a continuous basis. We want to raise the bar higher each year, highlight current problems and areas of weakness, and provide nationwide targets that are internationally acceptable and of a gold standard.

Established in 2012, the IHFD allows for a large casemix of subjects including that of 843 patients to date and has been made possible by the ever important place of information technology within medicine, the Hospital In-Patient Enquiry (HIPE) portal, managed by the Healthcare Pricing Office, HSE. The IHFD is a collaborative venture backed by the Irish Gerontology Society and the Irish Institute for Trauma and Orthopaedic Surgery.

## 2. Methods

The IHFD is a clinically led, web-based audit, whereby data is collected through the HIPE system. Participating trauma units submit data on all patients aged sixty or older admitted to their unit following hip fracture. Epidemiological data is submitted as well as premorbid functional and cognitive status. Comorbidity and physical status preoperatively are determined using the American Society of Anesthesiologists (ASA) physical status classification [10].

The timeline of events in hospital, time to orthopaedic ward, time to surgery, and length of stay, is also recorded. In cases where surgery was not performed within 36 hours, a reason for delay is required, chosen from a prespecified list of potential reasons. Data provided on postoperative care includes access to physiotherapy, occupational therapy, specialist medical review, and prescription of bone protection medication.

Of the 16 eligible hospitals/trauma sites within Ireland registered with the IHFD, 15 are currently submitting data. This first report provides analysis of patients discharged from 1 April 2012 to 31 March 2013. Only hospitals with at least 25 cases were included. Data submitted by 8 trauma centres was eligible for analysis. Each site had appropriate volume of hip fracture cases ranging from 25 to 226. Submitted data was 92% complete. Results presented as “not known” include data recorded as “unknown” or “not documented” as well as blank data fields. Two readmission episodes, deemed inappropriate, were excluded due to poor quality data. A total of 843 cases were included in the analysis.

## 3. Results

### 3.1. Baseline Characteristics

**Figure 1:**
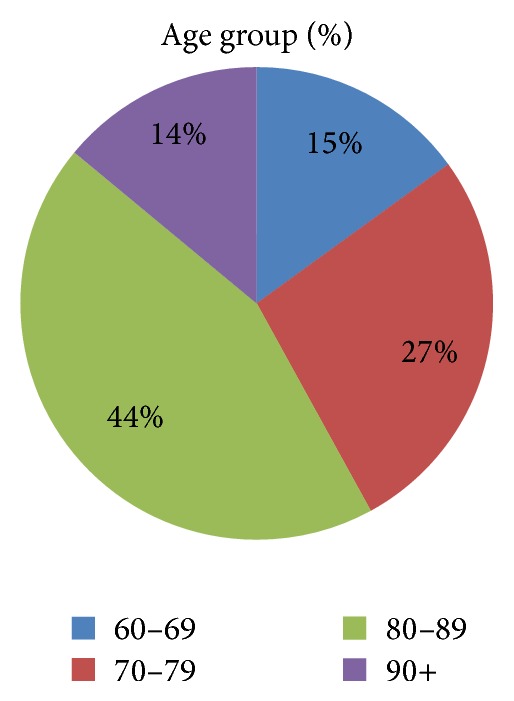
Age profile.

**Figure 2:**
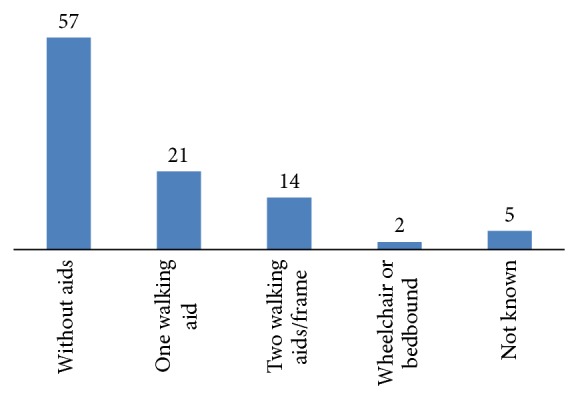
Prefracture walking ability %.

The majority of patients were female (70%) (Table 1) with more than half of all cases occurring in those aged greater than 80 years (Figure 1). There was a high percentage of comorbidity amongst the patient population with 44% of patients classed as ASA grade 2 (mild systemic disease) and 39% as ASA grade 3 (severe systemic disease that limits activity but is not incapacitating). No patients were deemed ASA grade 5 that is moribund with life expectancy of less than 24 hours with or without surgery. Despite the level of comorbidity, over two-thirds of patients were admitted from their home and 482, 57%, were independently mobile before fracture (Figure 2).

### 3.2. Fracture Classification

**Figure 3:**
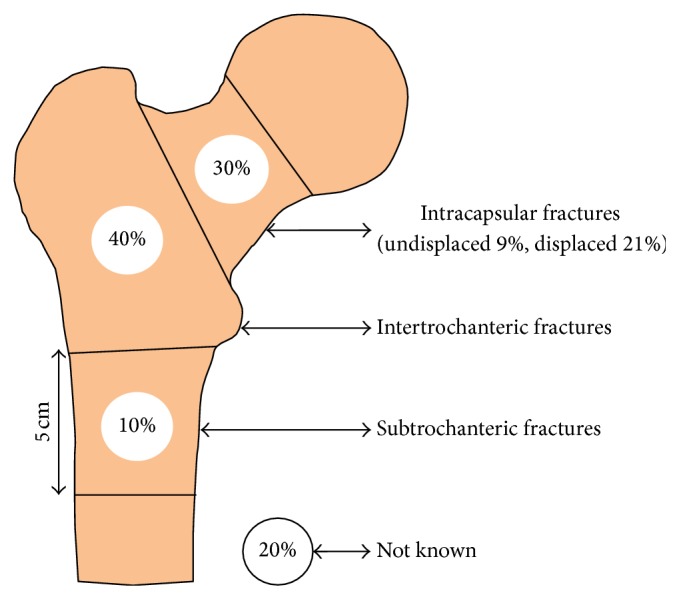
Type of fracture.

The majority of fractures were intertrochanteric, 40%. The remainder of cases were intracapsular, 30% (displaced 21% and undisplaced 9%), and subtrochanteric, 10%, with 20% not documented or unknown or blank (Figure 3).

### 3.3. Admission Details

676 patients (80%) were admitted via the emergency department (ED) at the operating hospital. Seventy-two percent presented directly to the ED with 5% transferred from another hospital. Data was not known for the remaining 3%. A further 20% were seen and diagnosed directly by the trauma team in the admitting hospital.

### 3.4. Admission Destination

The vast majority, 93%, of patients were admitted to an orthopaedic ward; however, only 28% were admitted within the Blue Book standard of 4 hours. Only 7% were seen routinely by a geriatrician preoperatively. A further 19% received a medical review on request and data was not known for 10%.

### 3.5. Surgery

Nine of the 843 patients did not undergo surgical repair of their fracture with data not known for a further 17. In all, 97% of the original cohort, *n* = 817, underwent surgery. One-fifth of cases were operated on outside “normal working hours,” that is, between 08.00 and 17.59, Monday to Friday. Time to surgery was not known for 27% of the surgical cohort. Of those with a known time to surgery, 60% of cases had undergone surgery within 36 hours of admission with 77% having surgical repair within 48 hours of admission. Reasons given for a greater than 36-hour delay in surgery included inability to access theatre (32%), awaiting surgical/medical review and/or stabilization of patients (38%), and inability to access a high dependency unit bed (3%).

In keeping with the dominant fracture type profile, extracapsular hip fracture, the most common operation performed, was internal fixation with a dynamic hip screw (DHS) in 34% while a further 28% underwent cemented hemiarthroplasty for a neck of femur fracture. Spinal anaesthesia, on its own, was most commonly used in 55% of cases. In a further 27% of patients, this was combined with general anaesthesia, 3%, or nerve block, 24%. In all, 20% were operated on under general anaesthesia.

### 3.6. Postoperative Care

Postoperative nursing care was good with only 4% of patients developing a grade 2 pressure ulcer or above during their admission. There was a failure to assess bone health or implement secondary fracture prevention measures in 25% of the cohort. Bone protection medication was commenced in 29% and a further 28% awaited either outpatient DXA scan or outpatient clinic assessment. Only 10% of patients were on treatment preadmission. An inpatient specialist falls assessment was carried out in 58% of cases with a further 4% awaiting outpatient assessment.

**Figure 4:**
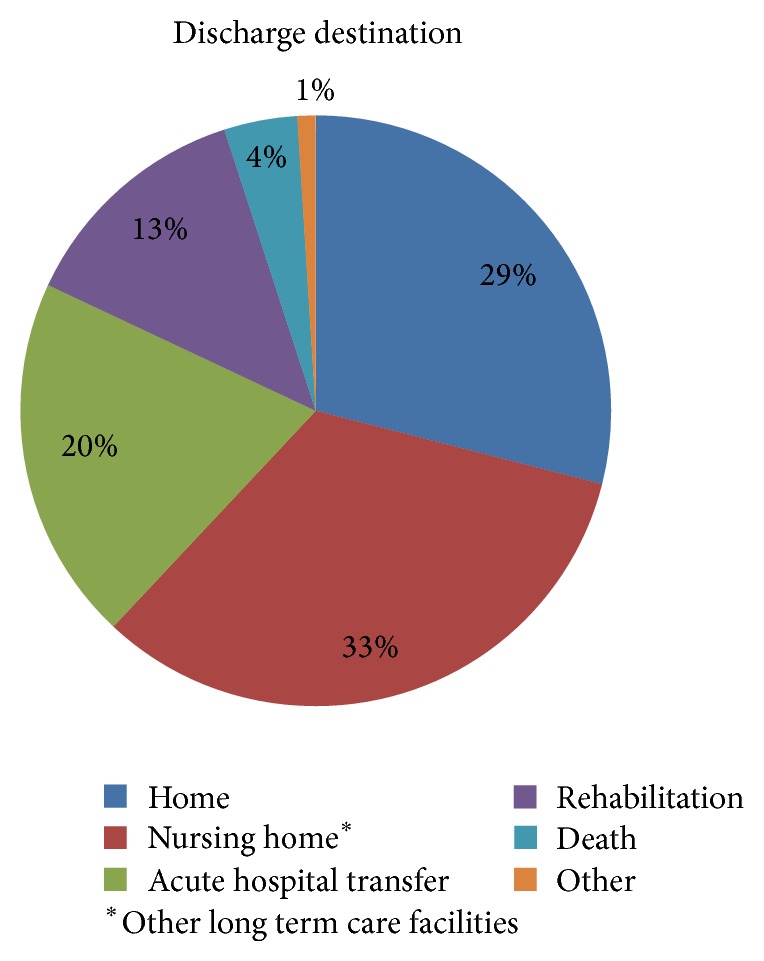
Discharge destination.

Despite 71% of patients being admitted from home, only 29% were discharged directly to home. One-third received ongoing care in a rehabilitation facility or transfer to another acute hospital. Although 10% of patients were admitted from a nursing home or long term care facility, 33% required discharge to a nursing home or other long term care facilities (Figure 4). The overall length of stay varied ranging from 1 day to 305 days with a median duration of 13 days. More than half of all patients had been discharged from the operating hospital within 14 days of admission (Table 2).

## 4. Discussion

Hip fractures are a major cause of morbidity and mortality in the older persons. The burden on healthcare providers is set to increase with improving life expectancy and an increasingly ageing population. It is estimated that there will be a 100% projected increase in the number of hip fractures for Ireland by 2026 [2].

In 2013, the Department of Health published healthcare quality indicators for the Irish health system which included two key performance indicators specific for hip fractures. The first was the 30-day mortality rate after hip fracture surgery and the second was the time to hip fracture surgery [11]. These together with the BOA-BGS Blue Book guidelines were the standards used in the IHFD.

The demographic data from the first year of implementation of the IHFD showing that female patients and patients in the 80–89-year age group are most likely to sustain a hip fracture from a fall at home is unsurprising and is similar to the data from the UK NHFD 2013 report [12]. The ASA grade was known for 89% of the patients with ASA grade 2 comprising the majority (44%) while in the UK NHFD the ASA grade 3 is the dominant group representing approximately 56% and ASA grade 2 comprised of 30%. A greater proportion of patients were independent ambulators (57%) compared to the UK NHFD (46%) with fewer using two aids or frame (14% versus 25%).

There were differences noted in the hip fracture pattern between the two databases. The fracture type was available in only 80% of patients. The dominant fracture type was intertrochanteric (40%) while in the UK NHFD it was the displaced intracapsular group (48%). A greater number of subtrochanteric hip fractures (10%) were reported in Ireland compared to the UK (6%). While some of these differences are likely due to the missing or incomplete data, it may potentially reflect a different trend in hip fractures in Ireland.

The majority, 676, of cases were admitted via ED in the operating hospital—72% came directly and 5% came indirectly via another, that is, the first presenting, hospital. The other, 167, cases were seen and diagnosed by the trauma team in the operating hospital and these included transfers directly from another hospital. While the majority, 93%, were admitted to an orthopaedic ward, only 28% were admitted within 4 hours; this is significantly less than the 50% reported in the UK NHFD (Table 3). In Ireland a cohort of patients will go directly to the operating theatre from the ED; therefore, admission to the orthopaedic ward is much later than the standard 4 hours. This is not accounted for in the data capture and may in part explain the large percentage of delayed admission to an orthopaedic ward.

While 57% were operated upon within the 48 hours, only 37% of these were operated upon within the normal working hours compared to 85% in the UK. The remaining 20% were operated upon outside of the normal working hours. In the UK the Best Practice Tariff initiative offers additional payment to the hospital that meets the Blue Book criteria as well as time to surgery of 36 hours. Surgery was performed within 36 hours in 44% of cases compared to more than 71% in the UK. The sizeable 27% of patients who did not have time to surgery recorded does not allow for accurate interpretation of this data. Similar to the UK NHFD the two leading causes for delayed surgery are the medically unfit patients awaiting review, investigation, or stabilization (35%) and awaiting theatre space (26%). There was a sizeable 19% with no cause recorded. The effects of delayed surgery in hip fracture patients cannot be overemphasized. A delay of more than 24 hours has been shown to increase the mortality rate [13, 14]. Furthermore, timely surgery reduces preoperative pain, the risk of developing decubitus ulcers, and medical complications and has been shown to reduce the length of stay [15–17]. It has been shown that the 30-day mortality risk is 2.5 times higher when surgery is delayed in those with medical comorbidities [18].

Only 7% of patients had been routinely reviewed by a geriatrician preoperatively. The involvement of an orthogeriatrician in the care of hip fracture patients has been shown to not only improve the morbidity and mortality in these patients but also reduce the delay in surgery, the length of stay, and the readmission rates [19–21]. It is important to note that at the time of submission of this paper for publication there was only one orthogeriatrician appointed in Ireland. Table 3 lists the Blue Book standards comparing the IHFD data to the UK NHFD data. To make the data comparable, percentages in the IHFD data are based on the exclusion of the “not known” data.

In terms of anaesthesia most patients, 79%, had spinal anaesthesia and 20% had general anaesthesia while 3% had a combination of both. In the UK approximately 48% had general anaesthesia and 38% had spinal anaesthesia and 5.7% had a combination of both. The most common type of procedure performed was a DHS (34%) followed by cemented hemiarthroplasties (28%) which is the most common procedure in the UK. As commented on earlier these differences are likely due to missing data but may reflect a different trend in hip fractures in Ireland. Open reduction and internal fixation with screws and total hip arthroplasties were uncommon at 3% each.

While only 4% had developed pressure ulcers a larger proportion, 6%, did not have this data recorded. The development of pressure ulcers is a difficult but preventable problem. Patients that develop ulcers in the postoperative period are known to have a higher mortality rate [22]. Specialist falls assessment took place in 62% of cases compared to the 94% in the UK (Table 3). These are areas that need improvement. The reported incidence of a second hip fracture is up to 16% within the first year postindex fracture; therefore, it is essential that all patients receive specialist assessment to prevent further falls and fracture [23–25].

Osteoporosis is one of the major risk factors for hip and other fragility fractures. In our study 10% of patients admitted with a hip fracture were on treatment for osteoporosis. A further 29% of patients were newly commenced on treatment during their hospital admission. While 28% were awaiting further assessment as an outpatient, 25% had not received any bone health assessment. It is reported up to half of hip fracture patients have already had one or more fragility fractures [26–28] and a recent meta-analysis has highlighted that 8.54% of hip fracture patients go on to have a second hip fracture with more than 30% of these within the first year [29]. Currently in the Republic of Ireland healthcare system assessment of fracture risk and treatment of osteoporosis is opportunistic in both primary and secondary care. Access to DXA is not routinely available and only a small number of the 16 trauma units receiving fractures have a dedicated fracture liaison service. The Irish Hip Fracture Database has highlighted, amongst other issues, the low rate of fracture risk assessment and treatment of osteoporosis within this study group both before and after the hip fracture. The information in this and subsequent reports will be used to influence and inform healthcare policy at a national and local level and focus resources on the improvement of outcomes for this high risk group of patients. The next IHFD report will be published in March 2015 and will report data from more than 2000 patients from 15 of the 16 trauma sites. An integrated care pathway for hip fracture patients, due for introduction in March 2015, will provide a framework to ensure all hip fracture patients receive appropriate preoperative and postoperative care.

The median length of stay was 13 days (range 1–305) with more than 58% having been discharged by 14 days. Despite 71% of patients being admitted from home, only 29% were discharged home with the majority, 33%, being discharged to a nursing home or other long term care facilities. The wide variation in length of stay likely reflects the different operating procedures for the discharge of patients in various acute trauma units, the access to local rehabilitation facilities, and access to state-funded long term care.

It is well known that patients with a hip fracture have a higher mortality risk [30]. There was a 4% inpatient mortality rate evident in our cohort. The UK NHFD report documents a 30-day mortality rate of 8.2% [12]; at present this is not being recorded in Ireland.

This preliminary report represents a starting point for the IHFD. We found inconsistencies with the interpretation of certain data points by the data collectors. These included geriatrician assessment, pressure ulcer development, specialist falls assessment, and bone health assessment. There were also incomplete or missing data, for example, AMTS score, which was only recorded in 58 patients. The interpretation of datapoints and the high rate of variables either not documented or not known is going to be addressed with continuous local and national validation of the data, development of a data dictionary, and education workshops for the data collectors.

Hip fracture is the most common serious injury of older people and also the tracer condition for the current global epidemic of fragility fractures. Hip fracture patients are usually older and frail and require thorough multidisciplinary input during both the acute and the rehabilitative phases of their care. As the numbers of hip fractures and subsequent costs rise, healthcare systems must develop integrated and systematic approaches to hip fracture care and secondary prevention of further falls and fractures.

In response to the challenges in the development and provision of hip fracture care, large-scale hip fracture audit has delivered measurable improvements in care and outcomes, including reduced mortality. The economic benefits to healthcare systems include reduced length of stay in hospital, reduction in further falls and fractures, and reduction in the need for long term care. The ultimate goal of the IHFD is to use data to drive the clinical and organisational improvements in quality, safety, and cost effectiveness of care and maximise outcomes for older people after their hip fracture.

## Figures and Tables

**Table 1:** Baseline characteristics.

Total *N* = 843	
Gender	
Female, *n* (%)	592 (70)
Age group, yrs Total, %	
60–69	15
70–79	27
80–89	44
90+	14
Source of admission, %	
Home	71
Nursing home^*^	10
Acute hospital transfer	18
Other	1
ASA^¥^ grade, %	
1	14
2	44
3	39
4	3
5	0
Premorbid functional status, %	
Independently mobile	57
One walking aid	21
Two walking aids/frame	14
Wheelchair/bedbound	2
Not known	5

^*^Plus other long-stay facilities; ^¥^American Society of Anesthesiologists.

**Table 2:** Postoperative care and Outcomes.

*Post-operative Care (n* = 809)	
Pressure ulcer, %	
No ulcers	91
Ulcers present	4
Not known	6
Bone health assessment, %	
Not assessed	25
Treatment commenced	29
Assessed, no treatment	2
On treatment before operation	10
Awaiting DXA scan	16
Awaiting OPD assessment	12
Not known	5
Specialist falls assessment, *n* (%)	
Assessed (inpatient/OPD)	499 (62)
No assessment	286 (35)
Not known	24 (3)

*Outcomes (N* = 843)	
Discharge destination *n* (%)	
Home	245 (29)
Nursing home^*^	277 (33)
Acute hospital transfer	172 (20)
Rehabilitation	108 (13)
Death	34 (4)
Other	7 (1)
Length of stay	
Median (range)	13 (1–305)
30-day reoperation (*n* = 817)	
Yes, *n* (%)	9 (1)
No	443 (54)
Not known	365 (45)

^*^Or other long term care facilities.

**Table 3:** Comparison of the first UK NHFD report in 2009 and the most recent UK NHFD report in 2013 with the first IHFD report.

Standard	UK NHFD 2009 preliminary report	UK NHFD 2013 report	IHFD 2013 preliminary report
(1) Admission to orthopaedic ward within 4 hours	N/A	50%	32%

(2) Surgery within 48 hours and during working hours	75%	86%	77%

(3) Patients developing pressure ulcers	N/A	3.5%	4%

(4) Preoperative assessment by an orthogeriatrician	24%	49%	8%

(5) Discharged on bone protection medication	N/A	69%	41%

(6) Received a falls assessment prior to discharge	44%	94%	60%
